# *Annals* Reports

**DOI:** 10.1111/nyas.12210

**Published:** 2013-07-08

**Authors:** Ten Feizi

**Affiliations:** The Glycosciences Laboratory, Department of Medicine, Imperial College LondonLondon, United Kingdom

**Keywords:** oligosaccharides, neoglycolipids, carbohydrate microarrays, carbohydrate ligands

## Abstract

Oligosaccharide sequences in glycomes of eukaryotes and prokaryotes are enormously diverse. The reasons are not fully understood, but there is an increasing number of examples of the involvement of specific oligosaccharide sequences as ligands in protein–carbohydrate interactions in health and, directly or indirectly, in every major disease, be it infectious or noninfectious. The pinpointing and characterizing of oligosaccharide ligands within glycomes has been one of the most challenging aspects of molecular cell biology, as oligosaccharides cannot be cloned and are generally available in limited amounts. This overview recounts the background to the development of a microarray system that is poised for surveying proteomes for carbohydrate-binding activities and glycomes for assigning the oligosaccharide ligands. Examples are selected by way of illustrating the potential of “designer” microarrays for ligand discovery at the interface of infection, immunity, and glycobiology. Particularly highlighted are sulfo-oligosaccharide and gluco-oligosaccharide recognition systems elucidated using microarrays.

## The diversity of oligosaccharide sequences in glycomes and the challenges for ligand discovery

Oligosaccharide sequences of glycoproteins, glycolipids, proteoglycans, and polysaccharides are enormously diverse. In mammalian glycomes, for example, they range from 2 to more than 200 monosaccharides in length joined by differing linkages, and some are modified, for example, by sulfation. Some oligosaccharide sequences are widely distributed in different cell types, whereas others are restricted to certain cell types; others are genetically determined (by glycosyltransferase genes). Examples of the latter are the blood group antigens and species-specific oligosaccharide antigens; compatibilities of these are major considerations in blood transfusion and xenotransplantation. Oligosaccharide sequences have a regulated expression at different stages of embryonic development and cellular differentiation, and they are often aberrantly expressed in cancer cells, such that they behave as differentiation antigens and cancer-associated antigens.^1^

The reasons for the diversity of oligosaccharides sequences within glycomes are unknown. There is an elegant hypothesis that the diversity may, in part at least, represent an evolutionary adaptation and diversification, under natural selection pressures in the course of endogenous and host–pathogen interactions.^2^,^3^ Indeed, there is increasing evidence that specific oligosaccharide sequences are directly involved as ligands in protein–carbohydrate interactions, *in vivo,* in health and, directly or indirectly, in the majority of diseases be they infectious or noninfectious. Examples of oligosaccharides as recognition elements in health include the folding of newly synthesized proteins in the endoplasmic reticulum, the routing of proteins (lysosomal enzymes) to their correct destinations in cells, the clearance of aged (asialo)-glycoproteins from serum, and the endocytosis of fungal pathogens for destruction. As discussed by authors in a recent focus issue on glycobiology of the immune response, (*Ann. N. Y. Acad. Sci*. **1253:** 1–221) protein–carbohydrate interactions have crucial roles in the regulation of inflammatory processes and in mechanisms of immunity (innate and acquired). Notable examples of oligosaccharides as ligands in disease processes are the attachment of adhesive proteins of pathogens to selected oligosaccharides of host cells at initial stages of infection.^4^ This is doubtless the tip of the iceberg.

With screening technologies such as carbohydrate microarrays, entirely novel and unsuspected carbohydrate recognition systems are being discovered, as for example, recognition of di-glucosyl-high-manose *N*-glycans by the endoplasmic recognition protein malectin.^5^ Another observation from microarray screening analyses for Simian Virus 40 (SV40) recognition was the finding that that the *N*-glycolyl analogue of ganglioside GM1 is preferentially bound.^6^ Unlike the *N*-acetyl analogue of GM1 ganglioside, which is found in humans and many mammals, the *N*-glycolyl GM1 is not synthesized by humans and is characteristic of simian species and other nonhuman mammals. The paucity of this ganglioside in humans may indeed be an example of evolutionary genetics of the type discussed above whereby humans have evolved not to synthesize *N*-glycolyl analogue of *N*-acetylneuraminic acid, and thereby develop at least a partial barrier to infection.

Identifying and determining the sequences of oligosaccharides that are ligands in biological systems that operate through carbohydrate recognition has been a challenging aspect of cell and molecular biology; this is because oligosaccharides cannot be cloned, they can generally be accessed in very limited amounts; and the affinities of interactions with recognition proteins are in most cases low. This review is focused on a technology, the neoglycolipid (NGL) technology, first introduced with my colleagues in 1985 in order to be able to carry out microscale direct-binding studies using oligosaccharides derived from glycoproteins.^7^,^8^ Here, I describe briefly the technology, the contributions before and following its miniaturization, and conversion to a state-of-the-art carbohydrate microarray system that is poised for ambitious glycomic analyses of carbohydrate recognition, and I dwell on some observations at the interface of immunity and glycobiology.

## NGL technology

### The impetus for developing the NGL technology—the principles

In classical studies that led to assignments of the structures of the major blood group antigens A, B, and H, the amounts of purified oligo-saccharides needed were enormous. These were hemagglutination-inhibition or inhibition-of-pre-cipitation assays requiring milligram mounts of purified oligosaccharide per well or per tube.^9^,^10^ The tricks of the trade at that time were to identify abundant sources of the antigens, such as the blood group antigen–rich mucin glycoproteins from ovarian cystadenomas. Similarly, these mucins served as sources of oligosaccharides, to characterize carbohydrate differentiation antigens such as the murine stage specific embryonic antigen, SSEA-1^11^ and the human myeloid cell-specific antigen,^12^ now termed CD15. The large amounts of oligosaccharides required in these types of analysis meant that ligands for very few other recognition systems involving the sugar chains of glycoproteins could be readily tackled.

Clearly, there was a need for a microtechnique, and this was the impetus for developing the NGL technology, which involves microscale conjugation of oligosaccharides via their reducing ends to a lipid molecule (Fig. S1). We selected an aminophospholipid and reductive amination as the conjugation principle. The resulting NGLs, with their amphipathic properties could be immobilized on matrices in a highly desirable, clustered state for direct-binding analyses. Moreover, the NGLs derived from oligosaccharide mixtures lent themselves well to binding experiments after resolution on thin layer chromatograms, similarly to glycolipids.^13^,^14^ The NGLs were found to have excellent ionization properties in mass spectrometry, thus the technology was elaborated to include chromatogram-binding experiments in conjunction with mass spectrometry^15^ (Fig. S2). The reductively generated NGLs serve well with oligosaccharides on which the recognition motifs are at the periphery of the chain. However, the core monosaccharide is largely sacrificed due to ring opening during conjugation to lipid. An alternative conjugation by the amino-oxy principle results in NGLs with a significant proportion of ring-closed cores^16^ (Fig. S1); this is advantageous for short oligosaccharides. Moreover, this also caters for recognition systems where, for example, the *N*-glycan core monosaccharide is involved as a part of the recognition motif.^16^ Although the NGL technology was originally established for oligosaccharides derived from glycoproteins, it is equally applicable to those derived from diverse glycoconjugates or synthesized chemically.^15^

### Contributions of NGL technology (1985–2002) before miniaturization for microarrays

**Figure 1:**
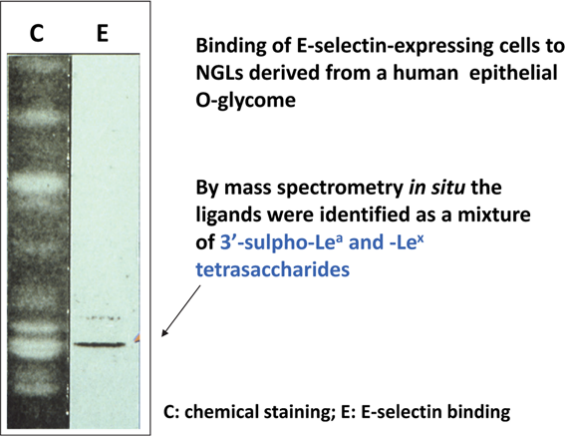
Oligosaccharide ligand discovery for the endothelium-leukocyte adhesion molecule E-selectin in a human epithelial *O*-glycome (adapted from Ref. 17).

An example of the power of the NGL technology for pinpointing ligands within glycomes is the discovery of sulphated Lewis^a^ and sulphated Lewis^x^ as ligands for E-selectin within the highly heterogeneous *O*-glycome of an epithelial mucin^17^ (Fig. 1). Other contributions (Fig. S3) included elucidations of differentiation antigens: the neural induction antigen L5 (Ref. 18) and the scrapie lesion antigen 10E4 (Ref. 19), and application of the technology in bacterial adhesion studies.^20^ The hitherto unknown *O*-mannosyl glycans were shown to be the sole *O*-glycan carriers of the HNK-1 antigen in the brain^21^ and to constitute about 30% of *O*-glycans in the brain.^22^ We have reviewed elsewhere the assignments of ligands for endogenous carbohydrate-binding proteins that are receptors of the immune system, enabled by the NGL technology^17^,^23^–^39^ they are also given in (Fig. S3). A remarkable cooperativity with effective display of the two ligands of P-selectin: sialyl-Le^x^ and sulfotyrosine in lipid-linked form on liposomes was also demonstrated.^40^

A highlight was the assignment of high-mannose *N*-glycans at Asn-917 of the complement glycoprotein, C3, as ligands for conglutinin (the first known C-type mammalian lectin).^26^ Conglutinin does not bind to the native C3 molecule. Only after two small peptides are cleaved off from C3 in the complement cascade, can conglutinin bind to the resulting glycopeptide iC3b (Fig. S4). A marked conformational change is known to occur after the proteolytic cleavages. These findings highlighted the profound influence of the polypeptide on glycan presentation for recognition; furthermore they demonstrated that commonly occurring carbohydrate chains can mediate biological specificity in particular body compartments.

## A carbohydrate microarray system based on the NGL technology—poised for deciphering the *meta*-glycome

### A state-of-the-art carbohydrate microarray system based on the NGL technology

Among special features of the NGL technology are that it is applicable to minute amounts of starting oligosaccharides,^41^ and analyses with glycosylceramides can be performed in parallel with NGLs.^8^ The technology lends itself well to generating designer microarrays from targeted sources, for example, from polysaccharides following their partial depolymerization,^42^ and from *N*- and *O*-glycomes,^43^ and in principle from the *meta*-glycome (a term I use, with instigation from Bernard Henrissat, to mean glycomes of diverse origins).

**Figure 2:**
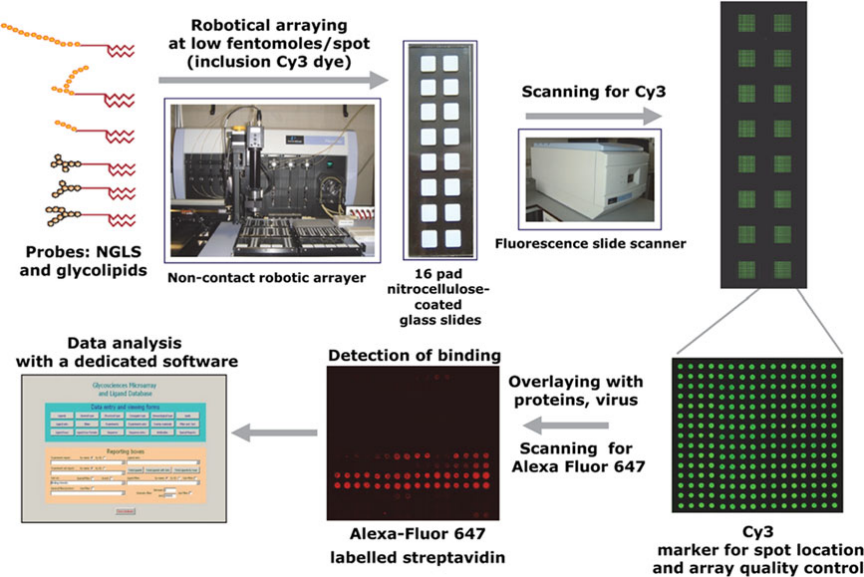
The neoglycolipid (NGL)-based microarray platform.

In 2002, we introduced an oligosaccharide microarray system based on the NGL technology.^44^ Following development, this is now a state-of-the-art system (Fig. 2).^42^,^45^,^46^ Our library of sequence-defined probes currently numbers ∼800, and encompasses a variety of mammalian type sequences, representative of *N*-glycans (high-mannose-type and neutral and sialylated complex-type), peripheral regions of *O*-glycans; blood group antigen-related sequences (A, B, H, Lewis^a^, Lewis^b^, Lewis^x^, and Lewis^y^) on linear or branched backbones and their sialylated and/or sulfated analogs; linear and branched poly-*N*-acetyllactosamine sequences; gangliosides, oligosaccharide fragments of glycosaminoglycans and polysialic acid. The arrays also include designer oligosaccharide probes of microbial and plant-derived homo-oligomers of glucose and of other monosaccharides (Fig. S5). Essential to the analysis system has been the development of a database for storage and scrutiny of microarray data.^47^ This holds all of the microarray data, the experimental conditions, and information on saccharide probes and proteins. There is an associated interactive software for presentation of microarray data, displaying data as tables, charts, or “matrices,” selective data retrieval, filtering, sorting, and deep mining of every data point.

### Contributions of NGL microarrays at the interface of immunity and glycobiology

Carbohydrate microarrays are revolutionizing the molecular dissection of protein–carbohydrate interaction.^45^,^48^ Salient ligand assignments made with the NGL technology since proof-of-concept microarray experiments in 2002^44^ are given in Figure S6. They include carbohydrate ligands for endogenous proteins,^5^,^42^,^49^–^53^ and the adhesive proteins of infective agents,^54^–^57^ as well as for neutralizing antibodies: antifungal^58^,^59^ and anti-HIV.^60^,^61^ I have selected below, by way of illustration, three topics at the interface of immunity and glycobiology.

## The gluco-oligosaccharide recognition by C-type lectin-like receptor Dectin-1

**Figure 3:**
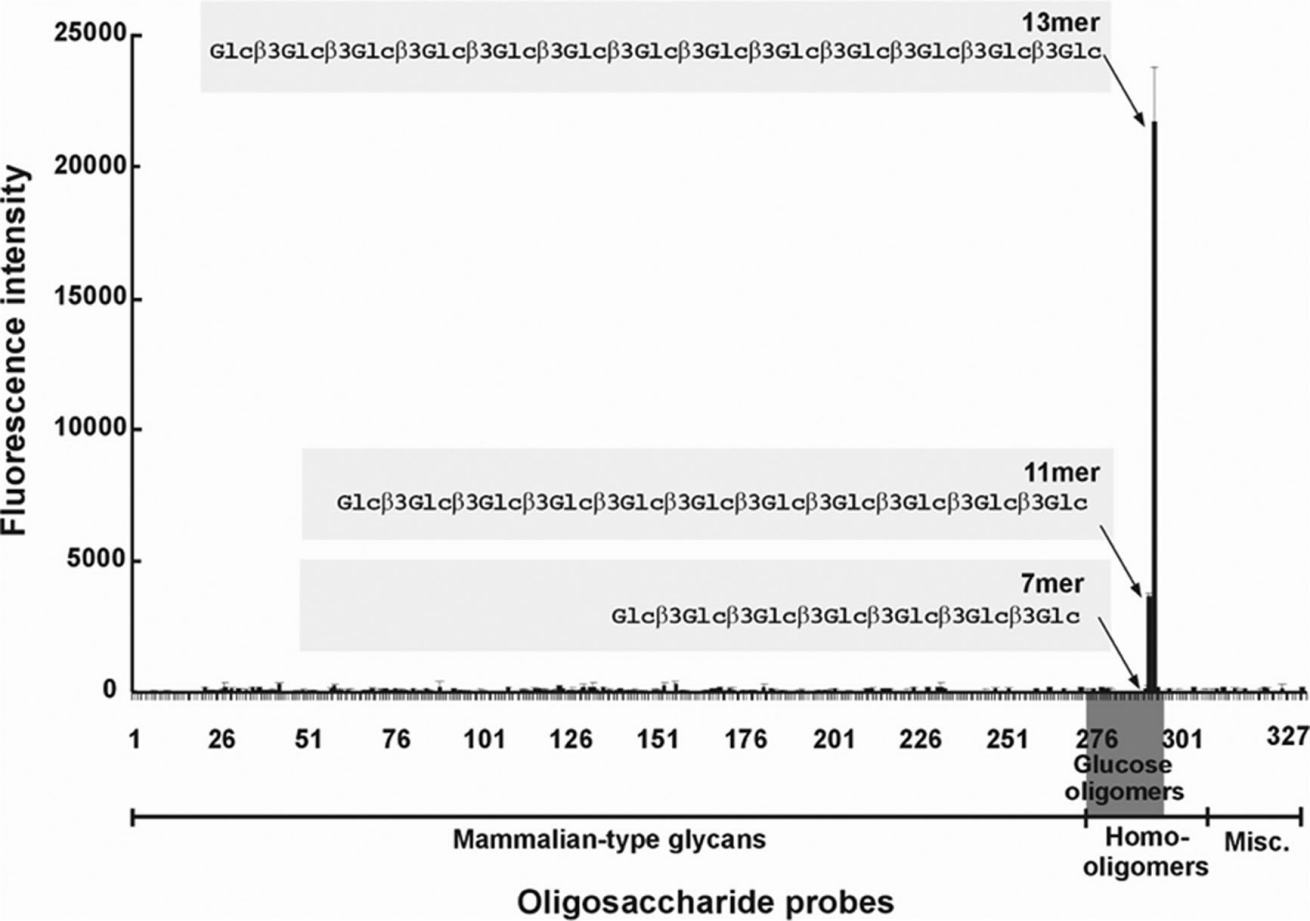
Microarray analysis of Dectin-1 showing binding only to β1,3-linked glucose oligomers >10 mers (from Ref. 45).

Dectin-1, the major receptor of the innate immune system against fungal pathogens is a C-type lectin-like protein on leukocytes.^62^ It has been reported to interact with a subset of T lymphocytes, but the main proven function for Dectin-1 is the mediation of phagocytosis and inflammatory mediator release in innate immunity to fungal pathogens. Although lacking in residues involved in calcium ligation that mediate carbohydrate binding by classical C-type lectins, Dectin-1 binds zymosan, a particulate β-glucan–rich extract of *Saccharomyces cerevisiae*; and binding is inhibited by polysaccharides rich in β1–3- or both β1–3- and β1–6-linked glucose.^63^ In order to prove that Dectin-1 binds to carbohydrates rather than to any polypeptides associated with the fungal cell walls and to identify the ligand structure(s), we generated designer probes, that is, NGLs, derived from oligosaccharides up to 13 mers isolated from partially depolymerized ligand-positive and ligand-negative glucan polysaccharides. These glucan probes were incorporated into microarrays.^42^ Upon probing the microarrays, the ligands for Dectin-1 were found to be unusually long, 10-mer or longer, β1–3-linked gluco-oligosaccharides. The β1–6-linked analogs were not bound (Fig. 3).^42^

The long chain requirement for Dectin-1 binding is apparent also with on-array inhibition experiments in which we evaluated activities of β1–3-linked 7, 11, and 13 mers in solution as inhibitors of Dectin-1 binding. Only the 11 and 13 mers were inhibitory (unpublished). Thus, the long chain requirement is not related to the mode of oligosaccharide presentation in the microarray, and points to the requirement for a conformational epitope, formed by the long β1–3-linked chains, analogous to the conformational epitopes on polysialic acid chains.^64^ This contrasts with data with other glucan recognizing proteins, such as antibodies (discussed later).^58^,^59^ These can bind short chains in the series. We followed up the biological significance, and observed that the ligand-positive oligosaccharides analyzed as NGLs on liposomes share the bioactivities of the intact polysaccharides.^42^ Thus far, microarray analyses using mammalian type glycans have not revealed an endogenous ligand for Dectin-1. Thus, the nature of an endogenous ligand believed to be expressed on T lymphocytes is an open question and will remain under review as new oligosaccharide probes become available.

Knowing that glucan polysaccharides can act as immunomodulators, we are broadening the repertoire of designer probes from glucomes to encompass all naturally occurring glucose linkages to study recognition by other carbohydrate-binding proteins of the immune system.

## The gluco-oligosaccharide determinants of vaccine-induced antifungal antibodies with therapeutic potential

The humoral response to glucans is another area of immuno-glycobiological interest that has potential for the development of therapeutic antibodies to improve the control of fungal infections in immunodeficient people. Anti-β-glucan antibodies elicited in mice by a laminarin-conjugate vaccine confer protection to mice challenged with major fungal pathogens such as *Candida albicans, Aspergillus fumigatus*, and *Cryptococcus neoformans*.^58^ A monoclonal antibody (mAb) 2G8 of IgG2b subclass has been selected for further study as it confers substantial protection against mucosal and systemic candidiasis in passive transfer experiments in rodents. The binding of this antibody to the β1,3-linked gluco-oligosaccharides is shown in Figure S7. Note that this antibody contrasts with Dectin-1, in showing binding signals with short oligosaccharide probes, as well as longer sequences.

Rather than opsonization of fungal cells, the mechanism of action of mAb 2G8 has been correlated with the antibody's targeting of the heterogeneous, polydisperse, high molecular weight cell wall, and secretory components of *C. albicans*. Two of these were identified as the GPI-anchored cell wall proteins Als3 and Hyr1. In addition, mAb 2G8 inhibited *in vitro* two critical virulence attributes of the fungus: hyphal growth and adherence to human epithelial cells.^58^ Data also suggest that the antivirulence properties of the mAb 2G8 antibody may be linked to its capacity to recognize β-glucan epitope(s) on some cell wall components that exert critical functions in fungal cell wall structure and adherence to host cells.^58^

Transfer of this antifungal mAb into the clinical setting would potentially enable the control of the most frequent fungal infections in immunocompromised patients. With this aim, two chimeric mouse-human derivatives of mAb 2G8, in the form of complete IgG or scFv-Fc, have been generated, transiently expressed in *Nicotiana benthamiana* plants and purified from leaves in high yields (approximately 50 mg/kg of plant tissues).^59^ Both recombinant antibodies fully retain the antifungal activities of the parent murine mAb against *C. albicans*, and like the parent antibody, they bind preferentially to the β1,3-linked glucan probes. Both the IgG and the scFv-Fc promote killing of *C. albicans* by isolated, human polymorphonuclear neutrophils in *ex vivo* assays, and confer significant antifungal protection in animal models of systemic or vulvovaginal *C. albicans* infection. Using new approaches to humanizing their *N*-glycans, these plant antibodies hold promise as therapeutic tools to confer protection against a broad range of fungal pathogens.

## Sulfo-oligosaccharide ligands: modulatory effects of sulfation on recognition by selectins, siglecs, langerin, and influenza viruses

As large numbers of oligosaccharides are probed in parallel and without bias, microarrays of oligosaccharides in glycomes, and of an increasing number of sequence-defined oligosaccharides, have a way of revealing hitherto unsuspected ligands. A recent unpredicted “hit” with mab AE3, which binds to diverse epithelial cancer tissues and to high molecular weight mucins that they shed, is the sulfoglycolipid SM1a.^46^ The carbohydrate sequence of SM1a has not been described on mucin glycoproteins. (We are pursuing the expression of the AE3 antigen on *O*-glycans in our Alliance of Glycobiologists NCI grant: http://glycomics.cancer.gov/teams/fukuda.)

An early example of an unpredicted finding within an epithelial *O*-glycome was the binding of the endothelial adhesion molecule, E-selectin to sulfo-oligosaccharide sequences of Lewis^a^/Lewis^x^ type (with sulfate rather than sialic acid) at the terminal galactose (Fig. 1).^17^ This showed that the combining site of E-selectin could accommodate equally well sulfate and the carboxyl group of sialic acid.^65^ The same was later shown for L- and P-selectins.^33^,^34^

**Figure 4:**
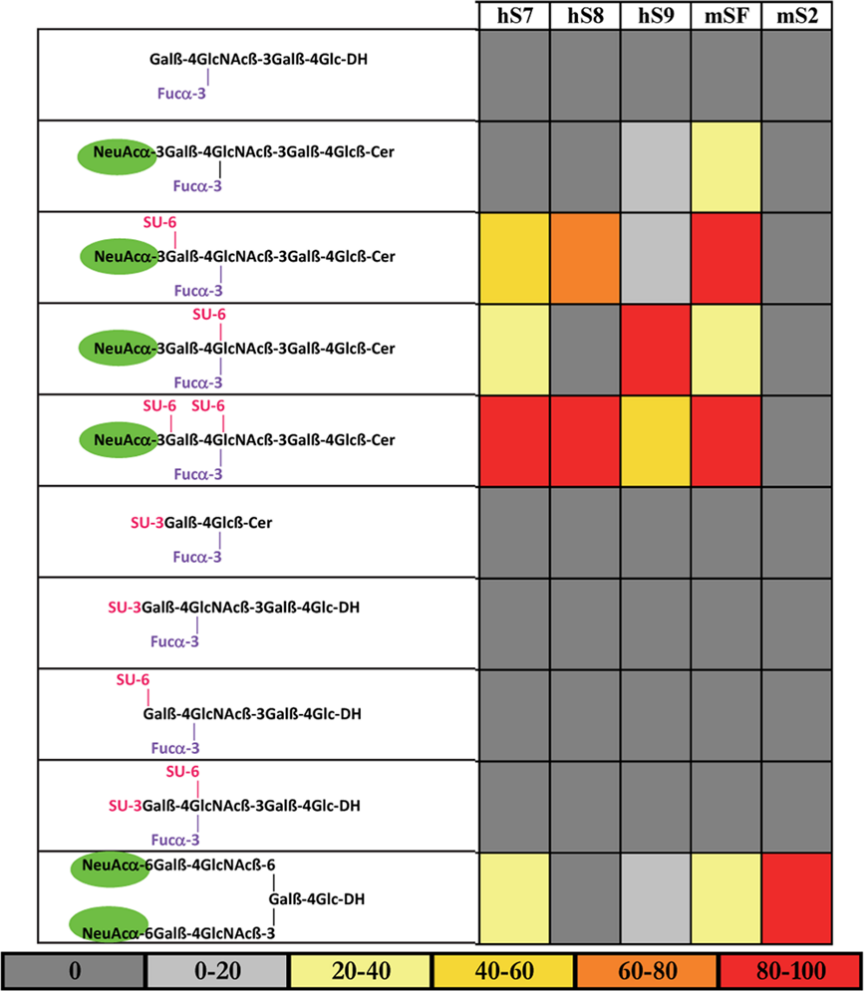
Microarray analyses of siglec IgG Fc chimera binding to sialyl and sulpho-oligosaccharide probes. Colors depict relative binding strengths calculated as a percentage of maximum binding to the best ligand for each siglec: three human (h) and two murine (m) (from Ref. 51).

**Figure 5:**
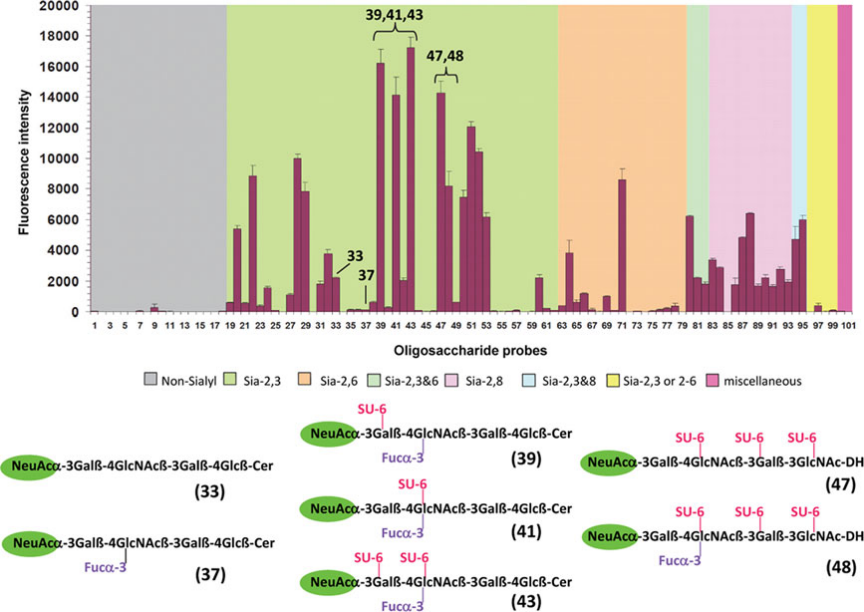
Microarray analysis of murine Siglec-E (from Ref. 53).

In the case of siglec recognition, however, microarray analyses have shown that the interchangeable role for sulfate and the sialic acid carboxyl group does not apply. As their name implies, siglecs absolutely require terminal sialic acid for binding (Fig. 4). Sulfate on sialyl oligosaccharide sequences can, nevertheless, modulate the strength of siglec binding.^51^ For example, the presence of sulfate 6-linked to the adjoining galactose or 6-linked to the inner monosaccharide, *N*-acetylglucosamine, can variously enhance or suppress interactions of the sialyl ligands of siglecs (Figs. 4 and 5). A detailed review of the modulatory effects of sulfation on oligosaccharide ligand recognition is out of the scope of this article. Suffice it to highlight that with well characterized, chemically synthesized glycolipids it was shown that with L-selectin, 6-linked sulfate at subterminal *N*-acetyl glucosamine (popularly termed 6-sulfo-sialyl-sialyl-Le^x^), enhances the binding to the sialyl-Le^x^ sequence and creates the preferred ligand for this selectin.^66^ This was corroborated by subsequent studies.^67^ In contrast, 6-linked sulfate at the galactose, 6′-sulfo-sialyl-sialyl-Le^x^, suppresses L-selectin binding while creating the preferred ligand for langerin.^49^,^66^

**Figure 6:**
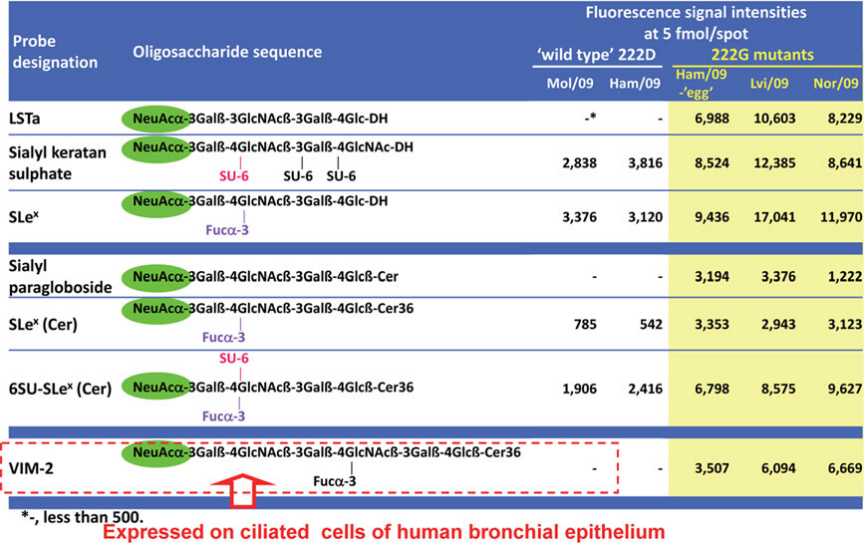
Highlights of microarray analyses of pandemic influenza A(H1N1) 2009 (pdm) viruses showing wild-type (222D) pdm viruses binding to α2,3 sialyl sequences with sulphate or fucose at penultimate *N*-acetylglucosamine, and mutant (222G) viruses increased binding to these and also binding to unmodified or internally fucosylated (VIM-2) sequences. The latter are known to be expressed on ciliated epithelial cells of the human bronchus (adapted from Refs. 56 and 57).

With the NGL-based microarray system, we have also observed an influence of sulfation of sialyl oligosaccharides on receptor-binding by the pandemic influenza A(H1N1) 2009 (pdm) virus.^56^,^57^ Although, as with seasonal influenza viruses, the strongest binding, overall, of the pdm viruses was to α2,6-linked sialyl sequences that are abundantly expressed in the upper respiratory tract, we detected binding of pdm viruses also to α2,3-linked sialyl sequences with sulfate (or fucose) at penultimate *N*-acetylglucosamine of the type that occur lower down in the respiratory tract (Fig. 6). The intensity of binding to these was enhanced with mutant viruses (D222G mutation of hemagglutinin) isolated from cases of severe or fatal pdm virus infection: with the D222G mutant pdm viruses there was binding also to nonsulfated internally fucosylated (VIM-2 antigen-bearing) sequences of the type expressed on ciliated cells of the bronchus (Fig. 6).^57^ The D222G mutant pdm viruses showed a change in cell tropism in cultures of differentiated human airway epithelial cells; they infected a higher proportion of ciliated epithelial cells than the wild-type pdm viruses. The additional binding of pdm viruses to α2–3 sialyl sequences that occur throughout the airway, including the lung, may account, at least in part, for the capacity of the pdm viruses to cause more severe disease than observed with seasonal influenza. These findings uncover potential mechanisms linking mutation D222G to severity of disease. If the mutant virus were to acquire the ability to spread more widely, the potential consequences would be of great significance, hence the need to closely monitor the evolution of this virus.

The various patterns of recognition of sulfo and fuco motifs by lectins of the immune system and the pathobiolgical consequences of the usurping of these by pathogens such as influenza viruses require investigation.

## An increasing variety of carbohydrate microarray systems

At the request of reviewers of the manuscript, I refer briefly to the considerable number of carbohydrate microarray systems that have been developed since 2002 using monosaccharides, polysaccharides, oligosaccharides, and glycolipids as starting materials. These and the diverse immobilization strategies used for the saccharides have been reviewed in detail.^45^,^48^,^68^–^76^ Among these are microarray systems with focused repertoires powerfully addressing specific recognition systems. In addition to our NGL-based system (http://www3.imperial.ac.uk/glycosciences), microarray systems with relatively large oligosaccharide coverage to screen for the specificities of diverse carbohydrate-binding systems are those of the Consortium of Functional Glycomics (CFG), Paulson, Cummings Bovin, and Gildersleeve reviewed in Ref 48; see also http://www.functionalglycomics.org/static/consor-tium/consortium.shtml. There is also the shotgun glycomics approach of Cummings and Smith for generating and probing microarrays from glycomes.^77^ Microarray analyses are variously generating an enormous amount of infomation on diverse carbohydrate-recognition systems. Without a doubt, glycobiologists will increasingly establish own microarray systems.

Clearly, the time is ripe to set up studies to formally compare the major microarray platforms that are being used to provide screening analysis data to the wide biomedical community. A cursory overview of results from the NGL-based microarray system and those from the CFG indicates that with robust carbohydrate-binding systems, there is good agreement. But there are differences that we are aware of, for example, the ability of the NGL system uniquely to detect binding of the 2009 pdm influenza virus (wild type), to α2,3 linked sialyl sequences.^56^ Are the differences a reflection of the analysis platforms or of the virus preparations used in the analyses? Platform comparisons need to be well orchestrated. The selection of recognition systems for the comparative studies needs to be based on a critical overview of existing data. This would form the basis for having preparations of particular proteins to share for the parallel analyses. There is also a need for standardization of experiments. This is now one of the missions of the project MIRAGE, which stands for minimum information required for a glycomics experiment (http://glycomics.ccrc.uga.edu/MIRAGE/index.php/Main_Page). The Interaction Analysis working group therein aims to define checklists for the standardization of experimental glycomics data and *meta* information.

## Concluding remarks

A decade after they were introduced, carbohydrate microarrays have come of age as essential tools in the exploration of glycomes for ligands of proteins involved in biological recognition systems. The data (i.e., the hits elicited) are informative and sometimes utterly unexpected. Microarrays are, of course, screening tools; they provide leads to be pursued to determine the biological significance of glycan-binding signals revealed. Without a doubt, microarrays are ideal tools for screening proteins in proteomes for carbohydrate-binding activities and assigning the oligosaccharide ligands within glycomes.

## Supporting Information

Figure S1 Preparation of probes as neoglycolipids (NGL) from reducing oligosaccharides by reductive amination or oxime ligation, for noncovalent immobilization on matrices: nitrocellulose, silica, plastics. **Figure S2**. The neoglycolipid (NGL) technology. **Figure S3**. NGL technology: contributions (1985–2002) prior to miniaturizing for microarrays. **Figure S4**. Schematic representation of complement glycoprotein C3 and derived glycoproteins and peptides. Approximate molecular masses of the α and β chains, and the fragments derived from the α chain following proteolysis in the complement cascade are indicated. Known glycosylation sites (filled lollipop), Asn-917 of the α chain and Asn-63 of the β chain, and a third nonglycosylated consensus site (unfilled lollipop), Asn-1595 of the α chain, are indicated (taken from Solis *et al*. 1994. *J. Biol. Chem*. **269:** 11555–11562). **Figure S5**. Composition of NGL-based microarrays. **Figure S6**. NGL-based microarray system: contributions since 2002. **Figure S7**. Dectin-1 and antibody 2G8-IgG binding to linear β1–3-linked glucose sequences derived from curdlan polysaccharide.
