# Supplementary material for: Annals Reports
Source: Ann N Y Acad Sci. 2013 Jul 8;1292(1):33–44. doi: 10.1111/nyas.12210 (PMC4260124; doi:10.1111/nyas.12210)
Supplement: Figure S1 — Preparation of probes as neoglycolipids (NGL) from reducing oligosaccharides by reductive amination or oxime ligation, for noncovalent immobilization on matrices: nitrocellulose, silica, plastics. Figure S2. The neoglycolipid (NGL) technology. Figure S3. NGL technology: contributions (1985–2002) prior to miniaturizing for microarrays. Figure S4. Schematic representation of complement glycoprotein C3 and derived glycoproteins and peptides. Approximate molecular masses of the α and β chains, and the fragments derived from the α chain following proteolysis in the complement cascade are indicated. Known glycosylation sites (filled lollipop), Asn-917 of the α chain and Asn-63 of the β chain, and a third nonglycosylated consensus site (unfilled lollipop), Asn-1595 of the α chain, are indicated (taken from Solis et al. 1994. J. Biol. Chem. 269: 11555–11562). Figure S5. Composition of NGL-based microarrays. Figure S6. NGL-based microarray system: contributions since 2002. Figure S7. Dectin-1 and antibody 2G8-IgG binding to linear β1–3-linked glucose sequences derived from curdlan polysaccharide. [file nyas1292-0033-SD1.pdf]

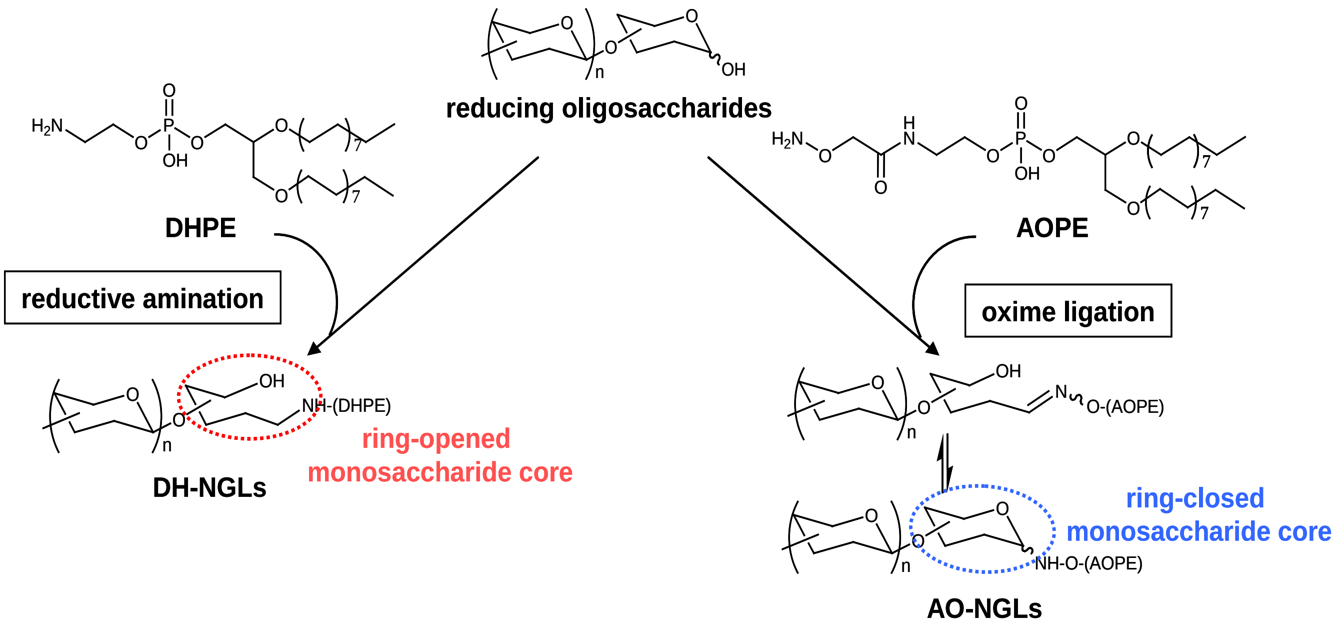

Supplementary Figure 1: Preparation of probes as neoglycolipids (NGL) from reducing oligosaccharides by reductive amination or oxime ligation, for non-covalent immobilization on matrices: nitrocellulose, silica, plastics.

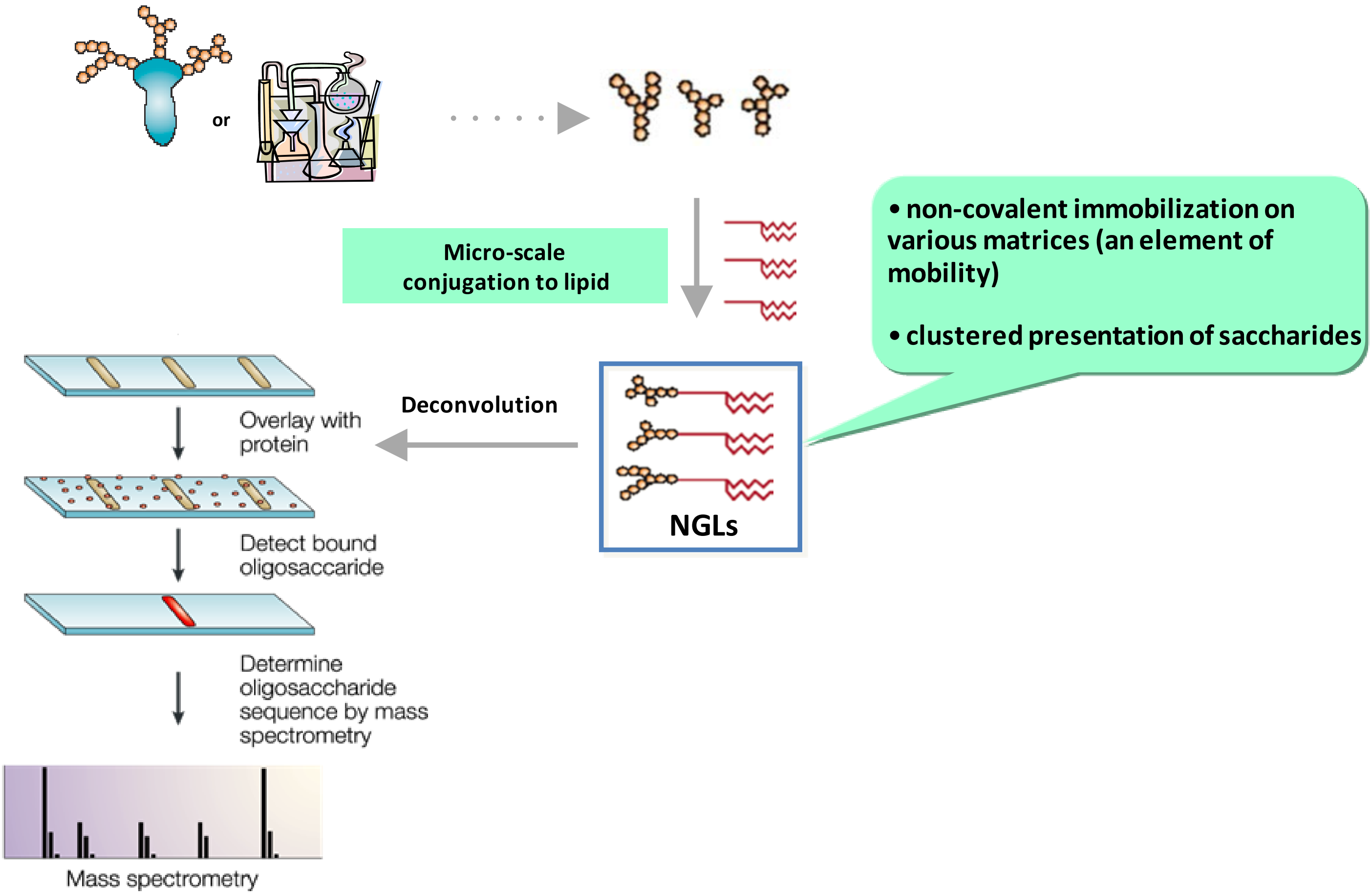

Supplementary Figure 2: The neoglycolipid (NGL) technology.

1985 –NGL technology
to study antigenicities and receptor functions of carbohydrate chains

Elucidation of carbohydrate differentiation antigens

Demonstration of carbohydrate receptors for bacteria

Contributions of NGL technology *O*-glycan sequencing

Discovery of *O*-linked mannose In the brain

Assignments of ligands for endogenous lectins

Conglutinin

Mannose binding proteins

Collectin-43
Pulmonary surfactant protein

Galectins 1 and 3

Amyloid P protein

Cysteine-rich domain of the macrophage mannose receptor

E-, L-, P-selectins

Supplementary Figure 3: NGL technology: contributions (1985-2002) prior to miniaturizing for microarrays.

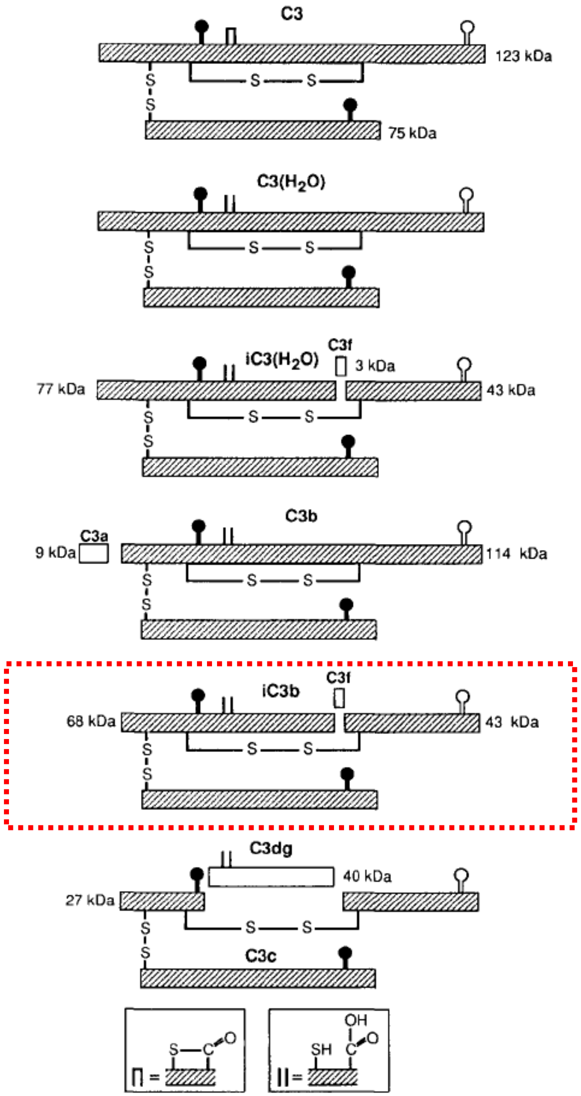

Supplementary Figure 4: Schematic representation of complement glycoprotein C3 and derived glycoproteins and peptides. Approximate molecular masses of the α and β chains, and the fragments derived fromthe α chain following proteolysis in the complement cascade are indicated.Known glycosylation sites (filled lollipop), Asn-917 of the α chain and Asn-63 of the *β*chain, and a third nonglycosylated consensus site (unfilled lollipop), Asn-1595 of the α chain, are indicated (taken from Solis et al. J. Biol. Chem. 269: 11555-11562. 1994)

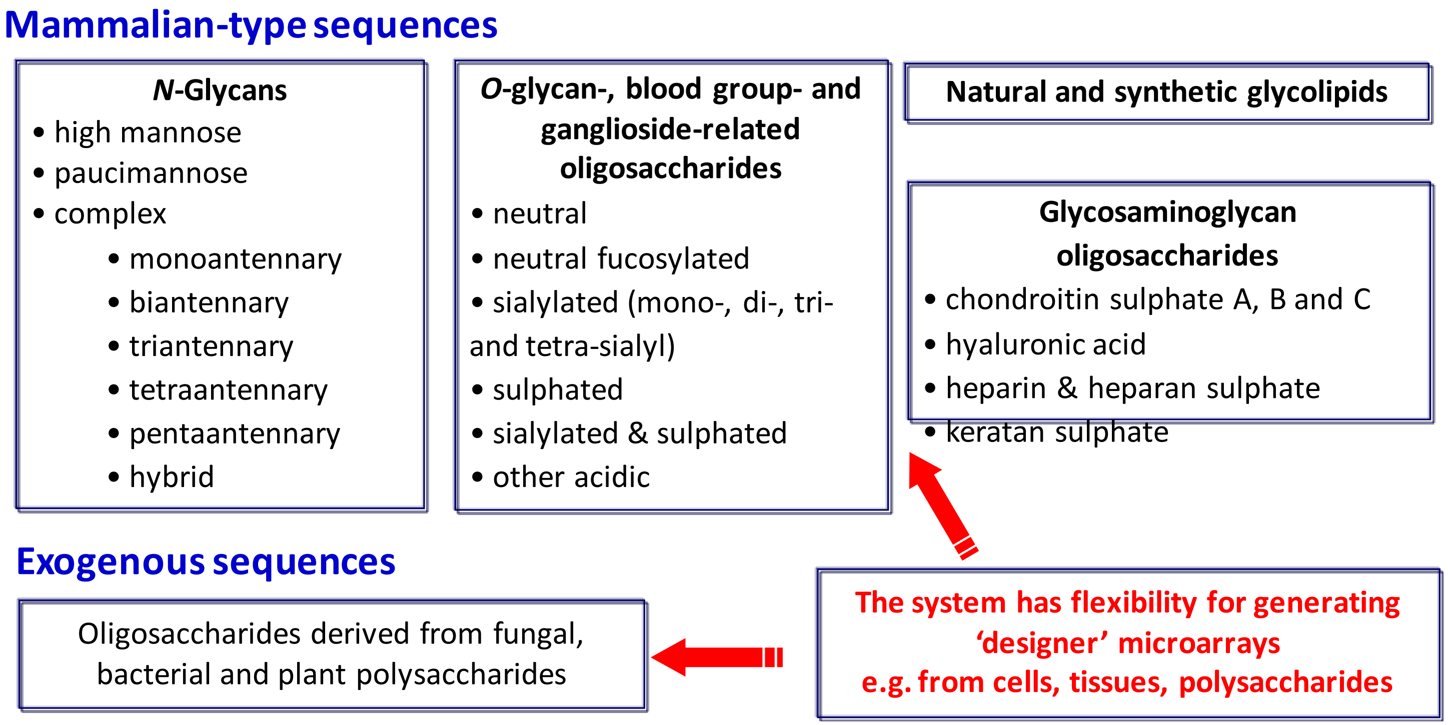

Supplementary Figure 5: Composition of NGL-based microarrays.

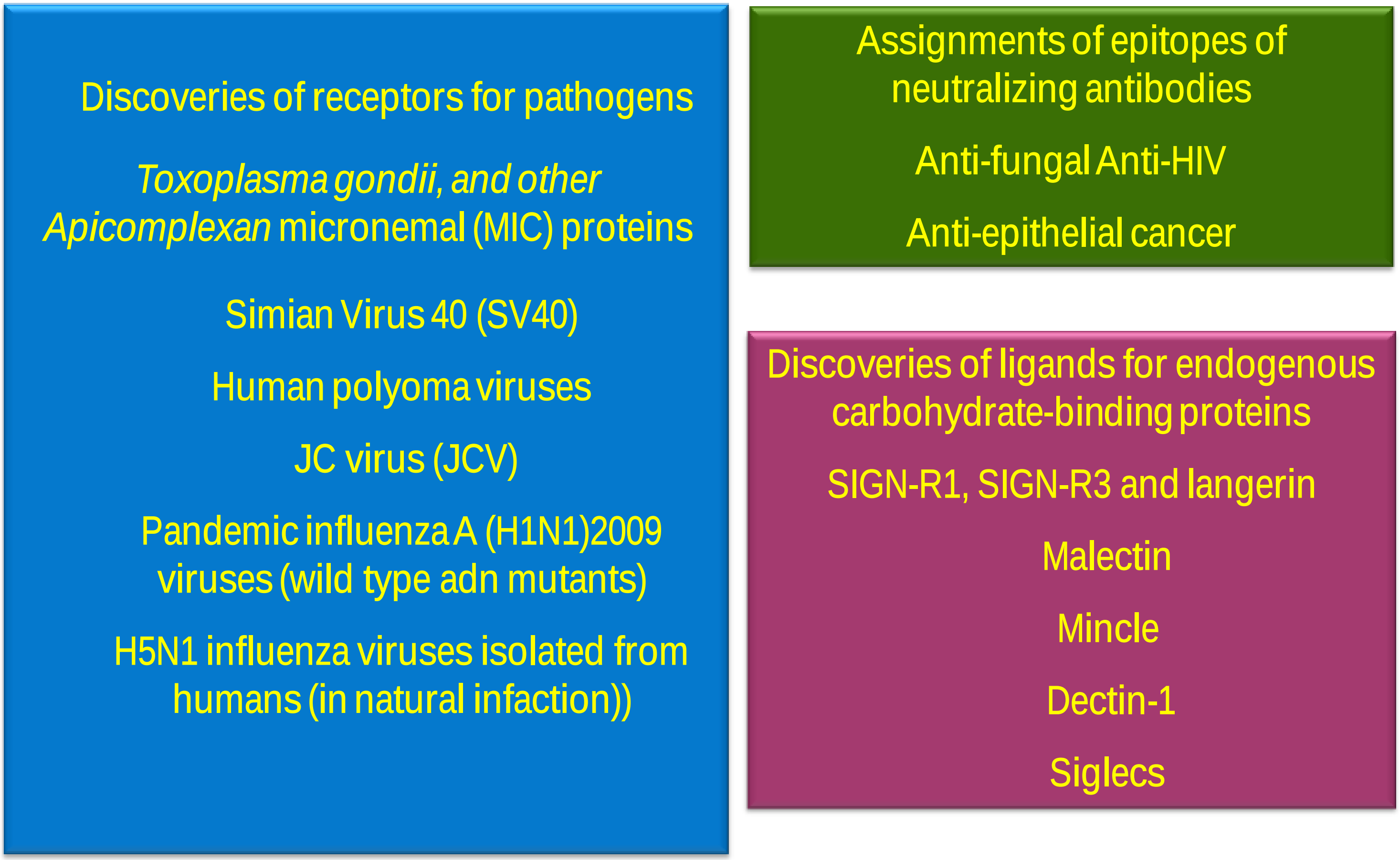

Supplementary Figure 6: NGL-based microarray system: contributions since 2002.

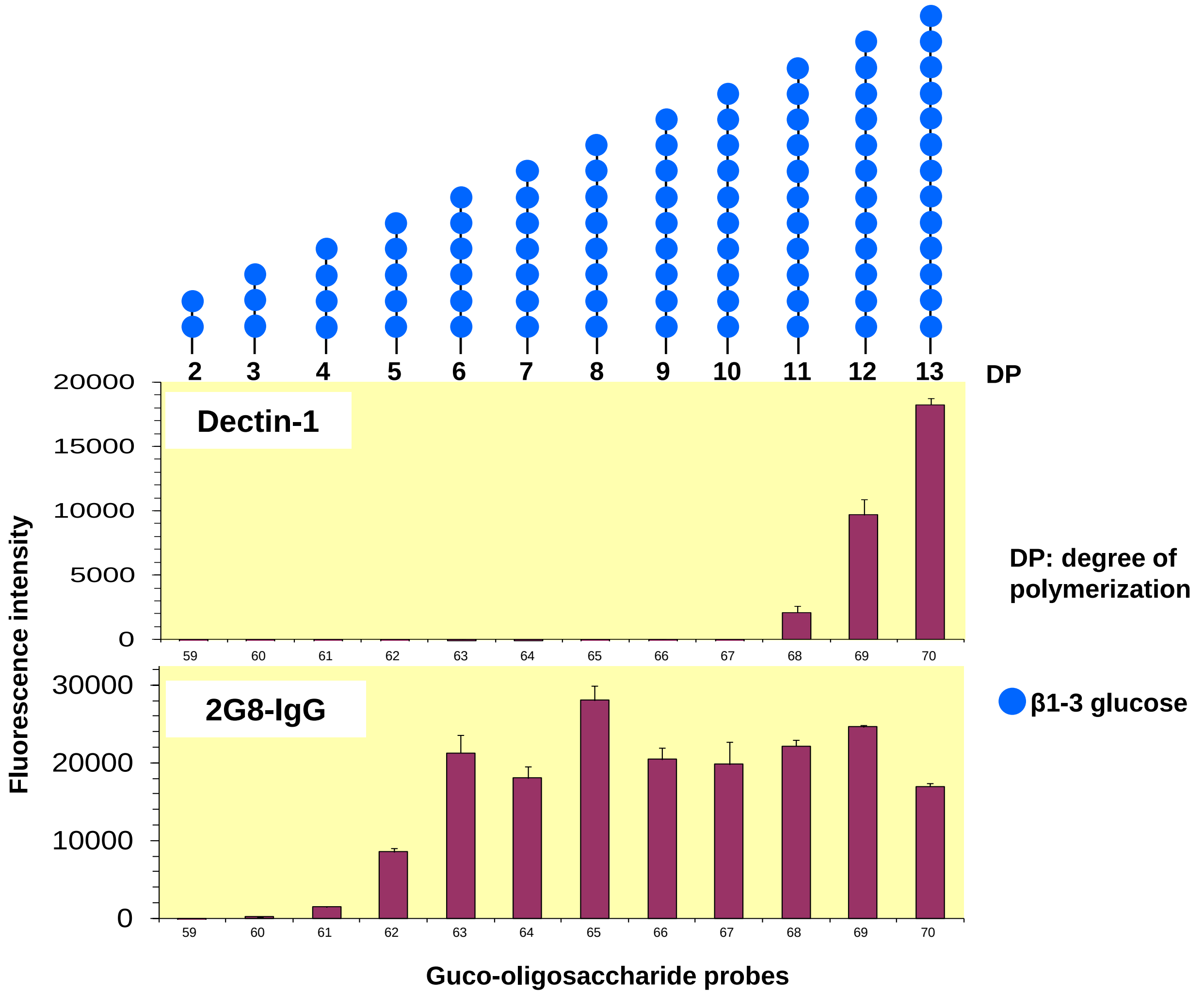

Supplementary Figure 7: Dectin-1 and antibody 2G8-IgG binding to linear β1-3-linked glucose sequences derived form curdlan polysaccharide.
